# Exploring women’s priorities for the potential consequences of a gestational diabetes diagnosis: A pilot community jury

**DOI:** 10.1111/hex.13036

**Published:** 2020-02-23

**Authors:** Rae Thomas, Anna Mae Scott, Rebecca Sims, Louise Craig, Leigh‐Anne Claase, Julia Lowe, Clare Heal, Leah Hardiman, Paul Glasziou

**Affiliations:** ^1^ Institute for Evidence‐Based Healthcare Bond University Gold Coast Qld Australia; ^2^ Therapeutic Guidelines Limited Melbourne Vic. Australia; ^3^ University of Newcastle Newcastle NSW Australia; ^4^ School of Medicine and Dentistry James Cook University Mackay Qld Australia; ^5^ Maternity Choices Australia Brisbane Qld Australia

## Abstract

**Background:**

There is no international diagnostic agreement for gestational diabetes mellitus (GDM). In 2014, Australia adopted a new definition and testing procedure. Since then, significantly more women have been diagnosed with GDM but with little difference in health outcomes. We explored the priorities and preferences of women potentially impacted by a GDM diagnosis.

**Method:**

We recruited 15 women from the Gold Coast, Australia, to participate in a pilot community jury (CJ). Over two days, the women deliberated on the following: (a) which important consequences of a diagnosis of GDM should be considered when defining GDM?; (b) what should Australian health practitioners call the condition known as GDM?

**Results:**

Eight women attended the pilot CJ, and their recommendations were a consensus. Women were surprised that the level of risk for physical harms was low but emotional harms were high. The final ranking of important consequences (high to low) was as follows: women's negative emotions; management burden of GDM; overmedicalized pregnancy; minimizing infant risks; improving lifestyle; and macrosomia. To describe the four different clinical states of GDM, the women chose three different labels. One was GDM.

**Conclusions:**

The women from this pilot CJ prioritized the consequences of a diagnosis of GDM differently from clinicians. The current glucose threshold for GDM in Australia is set at a cut‐point for adverse risks including macrosomia and neonatal hyperinsulinaemia. Definitions and guideline panels often fail to ask the affected public about their values and preferences. Community voices impacted by health policies should be embedded in the decision‐making process.

## 1 INTRODUCTION

Gestational diabetes mellitus (GDM) is a health condition diagnosed in pregnancy and associated with an increased risk of physical complications for the mother (eg caesarean section, pre‐eclampsia) and infant (eg macrosomia, shoulder dystocia).^1^ Women diagnosed with GDM have reported emotional and financial consequences such as self‐blame and guilt,^2^, ^3^ confusion over diet management^4^ and the expense of healthy eating.^5^ Other women reported the diagnosis as an opportunity to change their behaviours.^6^

The definition of GDM has a complex history. Definitions have used different theoretical premises (percentile range like most laboratory tests, the same values as in the non‐pregnant or risk based). Where a risk‐based assessment has been used, over time the focus has shifted. For example, in 1964, O’Sullivan and Mahan^7^ originally defined it as a way of identifying those women at risk of developing type 2 diabetes, whereas a large international study known as the Hyperglycemia and Adverse Pregnancy Outcome (HAPO) study^1^ in 2008 focused on foetal outcomes. In addition, the diagnostic test has varied^8^ from a 50 g glucose load 1‐hour screening test, a 75 g 2‐hour test and a 100 g 3‐hour test. Recommendations were initially for screening only ‘high‐risk’ pregnant women, but now include all women and diagnostic cut‐points have varied over several decades.^9^

The varying cut‐points arise because the risk of complications of gestational diabetes is on a continuum. The higher the mother's blood glucose level (BGL), the higher the risk of complications to her and her baby. The HAPO study^1^ aimed to find a cut‐point in the continuum to guide decisions on where the BGL should be drawn to define GDM. Unfortunately, an obvious cut‐point was not identified. Using the available HAPO data, the International Association of the Diabetes and Pregnancy Study Groups (IADSPG) consensus panel recommended a BGL threshold associated with the risk of adverse infant outcomes (such as risk of macrosomia, excess infant adiposity and neonatal hyperinsulinaemia).^8^ However, this change was controversial, and there is a lack of international consensus about the appropriate threshold between normal and elevated blood glucose.

Two recent Australian studies^10^, ^11^ compared the old GDM definition and testing regimen to the new. Sexton and colleagues^10^ in North Queensland reported an increase in GDM diagnoses from 10% under the old GDM definition to 20% under the new definition and Cade et al^11^ in Melbourne from 6% to 10%. Despite significant increases in the number of women diagnosed with gestational diabetes, both studies reported negligible health benefits for mothers and babies. The Cade study^11^ also reported a substantial increase in health‐care costs: a net cost increase for their hospital alone was over A$560,000 annually. Importantly, many (but not all, eg the Royal Australian College of General Practitioners) medical colleges in Australia adopted the IADSPG new threshold and testing regimen. Given the absence of consensus and the substantial increase in GDM diagnoses, we believe it is timely to explore the values and preferences of the women who may be directly impacted by the change in GDM diagnosis.

We conducted a citizen/community jury (CJ) to explore women's perspectives. A CJ is a form of deliberative democracy and aims to elicit an *informed* community perspective on important and often controversial topics. CJ participants are provided with expert presentations and opportunities to question the experts, engage in both facilitated and private deliberation and are asked to form a consensus or majority ‘verdict’ on the topic question. Community jury participants deliberate on questions requiring a decision that is both informed and ethically sensitive. CJs have been used successfully in research to elicit informed perspectives for several health policy issues, for example screening mammography,^12^ screening for prostate cancer,^13^ case finding for dementia,^14^ quantifying health preferences^15^ and more broadly in local governments.^16^, ^17^ We asked the CJ participants to deliberate on two questions:

Question 1: In the jury's view, which important consequences of a diagnosis of GDM should be considered by the consensus panel when discussing the Australian definition of GDM?

Question 2: What should Australian health practitioners call the health condition currently known as GDM?

## 2 METHOD

### 2.1 Steering committee

We established a steering committee to plan both the community jury and a consensus panel meeting to discuss the definition of GDM in Australia. The steering committee included a general practitioner, two endocrinologists, a consumer representative and two researchers with CJ and qualitative experience. This committee determined the eligibility criteria of the CJ participants, drafted the CJ questions and considered the expert information necessary for the jurors to make recommendations.

### 2.2 Participants

We recruited women from the Gold Coast region (Australia) and rural areas (identified via the Modified Monash Model^18^) within driving distance to the Gold Coast through Taverner Research, using social media advertisements and random generated landline and location sample drawn from SamplePages. CJ participants are sampled from the public most directly affected by the deliberation question; therefore, 15 women between age 30 and 45 years (median age of mothers for births registered in 2016 was 31 years^19^) were recruited. Women were eligible if they had had at least one pregnancy and self‐reported no previous diagnosis of GDM. We excluded women who had been diagnosed with GDM, were unable to provide informed consent and were unable to speak or understand English. A diagnosis of GDM impacts women in rural communities disproportionately than women living in urban areas^20^; therefore, we requested two women to be recruited from rural areas.

Once recruited, CJ participants were contacted by the research team and provided further information regarding organizational details of their attendance and informed consent documents to sign. CJ participants from urban areas were reimbursed $100 gift cards/d of the CJ. Women from rural areas were reimbursed $200/d and provided with overnight accommodation. Ethical approval was provided by the Bond University Human Research Ethics Committee (LC01914).

### 2.3 Procedure

The CJ was conducted over two weekend days, 2 and 3 February 2019, at Bond University (see Table 1 for schedule). All sessions, except for the private deliberation on Sunday, were facilitated by RT (a psychologist and experienced facilitator), who moderated discussions and ensured all jurors had equal opportunity to voice opinions. Two researchers, AMS, RS and an observer from Therapeutic Guidelines, LAC, observed CJ processes and jurors’ engagement for the sessions except the private deliberation. Only CJ participants were present during private deliberation to maintain confidentiality and prevent bias. Participants provided written consent prior to participating.

**Table 1:** Schedule of events

Saturday 2nd February		
9.00	Welcome, introductions, orientation to the CJ Why are we here?	Rae Thomas
9.45	What is gestational diabetes mellitus, how is it diagnosed, and what are the treatment options	David McIntyre
10.15	Q&A with David	
10.30	MORNING TEA	
10.45	A brief history of GDM definitions	Julia Lowe
11.15	Q&A with Julia	
11.30	The Australian context	Clare Heal
11.45	Q&A with Clare	
12.00	The potential consequences of a GDM diagnosis	Leah Hardiman
12.20	Q&A with Leah	
12.30	LUNCH	
1.00	Activity 1, 2	
3.00	End	
Sunday 3rd February		
9.00	Check‐in, reflections, orientation to today	Rae Thomas
9.30	Contact Experts	Rae Thomas
10.00	Activity 1 and 2 revisited	Rae Thomas
10.30	MORNING TEA	
Flexible timing	Deliberation	
	LUNCH	
	Present recommendations to Paul Glasziou (Chair of the steering committee and member of the consensus panel)	Paul Glasziou
3.00	End	

Four presentations were conducted on Saturday (via voice‐over PowerPoint) and followed with a telephone question and answer session with each presenter (descriptions of topics and experts are provided in Box 1). After the presentations, the CJ participants completed two activities (see below) to assist in answering the two jury questions.

BOX 1 **Expert presentations and download links**

1. What is gestational diabetes mellitus, how is it diagnosed and what are the treatment options?

Professor David McIntyre is an expert in obstetric medicine and a current Board member of the Australasian Diabetes in Pregnancy Society. He was a co‐author on the HAPO study.^1^

2. A brief history of gestational diabetes mellitus definitions

Associate Professor Julia Lowe is an endocrinologist working clinically with pregnant women. She was a co‐author on the HAPO study.^1^Professor Clare Heal is a general practitioner and researcher in rural Australia. She co‐authored an Australian publication of the prevalence and health outcomes of women diagnosed with GDM.^10^

3. The potential consequences of a gestational diabetes mellitus diagnosis

Leah Hardiman is a consumer representative and the past President of Maternity Choices Australia and represents women's experiences on national committees.All presentations can be viewed at: https://osf.io/mqd3c/?view_only=502b1cf9ef014e3589cb39b5d6c9d81b

On Sunday, CJ participants reconvened. Following a discussion about reflections from Saturday and overnight, participants were offered the opportunity to further question presenters for additional information and clarification. CJ participants elected a representative to facilitate private deliberation and deliberated on the two CJ questions until consensus or impasse was reached. The CJ recommendations were presented to the Chair of the Steering Committee Paul Glasziou and to the researchers and observers. At the conclusion of the CJ, participants were provided opportunity to debrief on CJ processes and outcomes.

### 2.4 Materials

#### 2.4.1 Presentations

Four experts provided presentations to the CJ participants. Presenters, their topics, a short biography and their presentation URLs are provided in Box 1. Participants were provided with biographies of the presenters and copies of PowerPoint presentations. No reimbursement (financial or otherwise) was provided to the experts.

#### 2.4.2 Activity 1: Ranking the consequences of GDM

The Steering Committee identified physical and emotional consequences often reported in GDM as important for the CJ participants. To quantify the physical consequences (eg pre‐term birth, caesarean section, macrosomia, shoulder dystocia), we used risk ratios identified by McIntyre et al^21^ to construct icon arrays for CJ participants. We conducted a systematic review of qualitative data^22^ to identify reports of emotional consequences. Twelve identified consequences were described on A4 size laminated paper and were presented and explained to the CJ participants. A further four blank consequence pages were also provided. A smaller version of each consequence and its description is provided in Supplementary File S1.

On Day 1, CJ participants individually ranked the consequences on a worksheet. The group then discussed their rankings and their reasons which were documented on a whiteboard. On day 2, the participants discussed their rankings again and deliberated in private.

#### 2.4.3 Activity 2: Considering the labelling of GDM

The facilitator (RT) presented a PowerPoint presentation of four scenarios representing four different clinical states/descriptions of how women might receive a diagnosis of GDM. The scenarios included a short story about a woman and a description of how she met the current Australian criteria for GDM. See supplementary File S2 for full example details.

The descriptions were as follows:

- Katie: developed diabetes as a result of pregnancy (ie this woman now met the criteria for non‐pregnant diabetes);
- Jenny: has higher than usual blood sugar levels as a result of the pregnancy and is at increased risk of complications;
- Emily: has higher than usual blood sugar levels as a result of the pregnancy and is at normal risk of complications; and
- Sofia: had diabetes before pregnancy and still has diabetes in pregnancy.

The risk of complications was deliberately varied in scenarios B and C to examine its impact on the label. See Supplementary File S2 for clinical descriptions provided to the CJ participants.

Participants were given scenarios and a list of the potential labels with two blank options describing the four different clinical states. The descriptors and the labels were developed in consultation with the Steering Committee, GPs and research colleagues.

On day 1, CJ participants were asked to identify their preferred label for each of the scenarios A–D followed by a discussion about reasons. CJ participants were asked to individually post each label to a laminated vignette which had been arranged on a wall. All labels had to be used, either with a vignette description or in a personal ‘discarded’ pile. Participants could also develop alternative labels. On day 2, CJ participants deliberated in private.

### 2.5 Statistical analysis

CJ recommendations were recorded on the whiteboard, and evidence of ranking and labelling was visually displayed and photographed. CJ proceedings were audio‐recorded and transcribed. CJ participant's recommendations to each question were presented at the end of day 2 and are provided verbatim. Two sections of the transcripts were qualitatively analysed to identify potential reasons for their recommendations (overnight reflections and private deliberations).

## 3 RESULTS

Taverner Research contacted 116 women for 15 to agree to participate (73 urban, 43 rural). Of the 15 women recruited, eight attended the CJ weekend: three failed to confirm attendance on the Friday before the CJ and failed to attend; three confirmed participants failed to attend without explanation; and one participant was unable to attend due to personal illness. One of the participants who did not attend was from a rural area. The participants’ mean age was 38 years; seven were married/defacto and one separated; all had children (1 with 1; 4 with 2; 3 with 3); four were born in Australia, two in New Zealand, one in the USA and one in the Philippines; one lived rurally and seven regionally.

### 3.1 Jury recommendations and potential reasons

#### 3.1.1 Question 1

*In the jury's view, which important consequences of a diagnosis of GDM should be considered by the consensus panel when discussing the Australian definition of GDM?*

##### CJ recommendation

The CJ participants grouped together some of the 12 consequences identified from the systematic review as they considered this best reflected their content. For example, the consequence labelled ‘burden of managing GDM’ also incorporated ‘dietary management‐related stress’ and ‘increased medical appointments’. Therefore, the list of 12 consequences was reduced to six. The women ranked the new groups of consequences that they considered the most important to women, from highest priority to lowest (see Box 2).

BOX 2 **CJ participants’ ranking of important consequences**

1. Negative emotions such as self‐blame, sadness and anxiety
2. The burden of managing GDM (including dietary management stress and increased medical appointments).
3. Over‐medicalized pregnancy (including caesarean sections)
4. The opportunity to minimize the risks to the baby (including shoulder dystocia or birth injury and pre‐term birth).
5. The opportunity to improve lifestyle (including reducing a women's risk of developing diabetes after pregnancy)
6. Having a ‘big’ baby

The CJ participants concluded that:

P4: We felt collectively that the most significant consequence of being diagnosed, being told you've got a diagnosis for women was the negative emotions that go with that, the guilt and the ‐ everything, self‐blame and sadness and dread and all that, expectation and all of that. We felt collectively that that was the number one consequence. (day 2 page 131)

The women did not identify any additional consequences to include than those synthesized from the available evidence.

##### Potential reasons from reflections and deliberations

At the start of day 1, the women had individually ranked the potential consequences of GDM on day 1, most women (5/8) ranked the baby's well‐being as top priority (eg ‘opportunity to minimize risks to unborn baby’):

P2: It's all about the baby

RT: …. Tell me what you mean by it's all about the baby?

P2: Make sure that baby is safe

PX: It's healthy. (day 1 page 58)

On day 1, the consequences related to the woman's well‐being (eg negative emotions such as self‐blame, sadness and anxiety) stemming from being diagnosed with GDM were a lower priority. However, views differed:

P6: I think all about the baby is just a kneejerk reaction. (day 1 page 58)

and a rigorous discussion followed about potential risks to mother and baby.

At the start of day 2, most CJ women reconsidered their original position on the consequences of a GDM diagnosis as a result of reflection and discussions with friends and family, for example:

P6: I talked to my husband about it last night and I spoke to my sister who's a midwife and texted my ‐ the couple of friends I mentioned who had gestational diabetes and yeah, just reflected on it, I suppose…. I felt like I did have insights, yeah…. I don't quite know how to word it, basically the risks of gestational diabetes are a lot less than I previously thought. (day 2 page 1)

P7: I had just a quick chat about it with my husband about it yesterday, just explain what we did, but we didn't really talk about it too much, but had it swirling around in my head trying to grapple with the 12, ordering the 12 most important and I had sort of negative emotions at number three and I think that is definitely moving up, but I'm still grappling with the numbers of risks for birth injury and that sort of thing… maybe those numbers [eg around birth injury] don't really matter because there's still such a small risk. So that's just what I've been thinking for the past 12 hours. (day 2 page 8)

Other women changed their minds after reflecting on the evidence that was presented by the experts on day 1 of the Community Jury.

P2: I kind of went from more focused on the minimised risk [to the baby], because obviously we didn't have the numbers in front of us… But now knowing that the risks are quite minimal in terms of numbers, …. the focus is more about the woman's wellbeing, her stress, her mental health… and we saw a few yesterday after that whole risk thing. We were like, oh okay, yeah, the risk is minimal, so I think there was a few of us going, our minds have turned a bit, because I know for me now it is, yeah, kind of pretty much flipped. (day 2 page 2)

The women also articulated the distinction between the instinctive and emotional and more deliberative thinking processes.

P4: Well our first reaction's always an emotional one, isn't it; we don't automatically look for numbers, we automatically react emotionally. (day 2 page 133)

The women recognized that because their views were based on evidence presented by the experts on day 1, they would likely differ from those unfamiliar with the evidence.

P2: So we are thinking from the point of view that we know all the information and that's why we're putting that ‘risk to baby’ down at number five… But someone who doesn't know that information, like we have, that's going to be quite high actually….

P6: That's why we're an informed community jury though, because we do know the numbers, we know that that's what makes it bump down. (day 2 page 105)

More specifically, having learned, for example that the increase in risks that are associated with a GDM diagnosis, is small for many outcomes, led to the women to consider that a diagnosis of GDM may not be helpful—and possibly even harmful—to some pregnant women:

P7: … that's something we need to really think about, that women that may have been fine without it [GDM diagnosis] may have been teetering on the edge of anxiety and that sort of thing.

P4: And maybe fine through the whole pregnancy. If they were at the very low end…

P7: Right, exactly

P4: …then there might be no ‐ there might be zero consequences. (day 2 page 82)

Similarly, having learned that the risks of a caesarean section differed between the diagnosed (24/100) and not‐diagnosed (17/100) women by only 7/100, also led the jurors to change their minds on its relative importance.

P1: I don't even think they should be F***ing told about the caesarean. I think it's…

P4: But the doctors have to tell them, it's a potential risk

P1: But that's what I'm saying, she said you can take them out. There's 100 potential risks and [Participant 7] has read it and it was no different, was it?

P7: No, there was actually a drop in the incidence of emergency caesareans in one study

P4: But you're right, we agree that them saying that they might have to have a C‐section is like putting the cast on a bruise, it's just a fearmongering things, it's just in case. (day 2 page 104)

#### 3.1.2 Question 2

*What should Australian health practitioners call the health condition currently known as GDM?*

#### CJ recommendation

The women's final recommendations and their reasoning for what to label the four different clinical states of GDM is described in Box 3 from *most preferred* to *least preferred* for each descriptor. The women did not identify any other potential labels for GDM than those suggested and provided by the Steering Committee and clinical and research colleagues.

BOX 3 **CJ participants’ preferred labels to describe the differing clinical states of GDM (in descending preference)**

- Katie: developed diabetes as a result of pregnancy (ie now meets non‐pregnant diabetes criteria)

*Gestational diabetes (most preferred)*
Pregnancy induced diabetes
Diabetes in pregnancy
Diabetes due to pregnancy (least preferred)

*P4: We're labelling Katie because we want her to have all the information and full assistance and make sure she knows exactly what she needs to do and go to the extra appointments and all the rest of it. And we didn’t know whether the outcome after her pregnancy would still be diabetes, we weren't 100 per cent sure, so we listed it this way, gestational, we thought we'd label her with gestational, pregnancy induced, diabetes in pregnancy and diabetes due to pregnancy. (day 2 page 136)*

B. Jenny: has higher than usual blood sugar levels as a result of the pregnancy and is at increased risk of complications

*Raised blood sugar in pregnancy (most preferred)*
Reduced tolerance to raised blood sugar in pregnancy
Altered glucose metabolism in pregnancy (least preferred)

*P4: So we decided that Jenny doesn't need to be told that she's got gestational diabetes because it would scare her to death and it's not necessary. We decided that we would label Jenny as having raised blood sugar in pregnancy, so that she's aware, she can do what she needs to do, but she's not panicking, she's not needing to go to 100 appointments and change too much significantly, be aware and make the necessary changes, et cetera.(day 2 page 136)*

C. Emily: has higher than usual blood sugar levels as a result of the pregnancy and is at normal risk of complications

*Raised blood sugar in pregnancy (most preferred)*
Pregnant
Reduced tolerance to raised blood sugar in pregnancy (least preferred)

*P4: …. we like Emily and we decided to also not label her as having gestational diabetes because we don’t think it's necessary, based on our list of consequences, we're trying not to freak her out, so we're going to tell her she's got raised blood sugar and give her all the information and try and get her through it that way without needing all that extra stuff. (day 2 page 137)*

D. Sofia: had diabetes before pregnancy and still has diabetes in pregnancy

*Diabetes (most preferred)*
Diabetes in pregnancy
Gestational diabetes
Hyperglycaemia in pregnancy (least preferred)

*P4:…. so she's diabetic. So we started this one first and got a bit excited, she's diabetic. She's also pregnant. She's got diabetes and pregnancy, gestational, so this was our list. (day2 page137)*

#### Potential reasons from reflections and deliberations

Some CJ participants reported overnight ruminations about the label GDM. For example,

P4: It's not labelled as a disease, but it feels like a disease, it's treated as a disease when it's a risk. (day 2 page 5)

P5: …. I just sort of like thinking about all the definitions and even the terminologies used or just referring to it as gestational and diabetes, I thought with the definition and the risk, it shouldn't even be called that. Like because for someone who is not ‐ who's never had diabetes or a new mum, it's pretty confronting, I thought, to be told that you've got it because you know that is a chronic illness …… (day 2 page 8)

Decisions on what to label the different clinical states of women diagnosed with GDM centred around balancing the emotional harms following a GDM diagnosis and providing women with a label that informed her of the seriousness of her raised blood sugar level. The women differentiated between the different clinical scenarios and grappled with labelling them all as GDM.

P4: Yes, so that's ‐ would you say what she's got, would you define ‐ if Jenny [scenario B] and Katie [scenario A], came in on the same day, would you tell Jenny the same thing that you would tell Katie?

P2: No

P4: So that's where we can't label them the same

They were more nuanced in their terminology and considerations of diagnostic impact than the current definition allows,

P7: Why wouldn't we just call this one gestational diabetes as it stands now?

P6: Yeah, I would, yeah

P7: Because I think the issue with the consequences of telling a woman that falls at the really low end of the spectrum, the consequences for her [one scenario] are significantly more than her [another scenario] because she needs to manage her condition…

[…]

P7: She needs the lower ‐ you don't need to jab yourself, we're not going to book you straight in for an induction and C‐section, but you need to be careful…

P2: Yeah

P7: …and monitor your blood sugars and we'll monitor you, maybe you'll have two extra appointments that a regular, normal, less risk, this lady's going to have six, somewhere in between and split the condition… (day 2 page 119 – 120)

and maybe more cautious,

P4: Poor all Katie [scenario A], sorry love

P5: We've just diagnosed you. (day 2 page 120)

The two scenarios with higher than usual blood sugar but different risks of complications (scenarios B and C) had the most discussion and the most concern about unnecessary harms. For example, when discussing the scenario depicting raised blood sugar with normal risk (scenario C), the women deliberated:

P4: Emily [scenario C] has higher than usual blood sugar levels as a result of the pregnancy and is at normal risk of complications. So she's never had it before, she's eating too much Baskin, how do we define her? Do we tell her she's diabetic, do we tell her she's…

P2: Well I think most things up there [potential labels posted on scenarios by women earlier] are related to blood sugar and pregnancy, so if we go by…

P4: So how do we label, because our job is to label what she's got

P2: I would tell her she ‐ I'd just tell her she's pregnant. I wouldn't tell her anything else

P4: So do we tell her she's ‐ so raised blood sugar and hyperglycaemia, is that not the same thing but one's a scary… (day 2 page 109)

While for scenario B with increased risk,

P7: But she's at ‐ she is at an increased risk of complications

[…]

P4: Keeping in mind what the complications are, which are minimal, how do we ‐ what do we ‐ how do we define her?

P2: Leave her alone….

[Laughter]

P6: Yeah, I say that we go raised blood sugar in pregnancy for her

[…]

P4: So we're just going to warn her that this is happening, if you continue this way, this is going to be the outcome… ……..So reduced tolerance to raised blood sugar, would that be ‐ yeah? So we're not going to tell her she's got pregnancy induced diabetes, that's down the bottom, right?

P6: Then I think we should get rid of diabetes limited to pregnancy and pregnancy induced diabetes for Jenny [scenario B]

PX: Me too

P4: Because we don't want to stress out poor Jenny [scenario B]

However, they recognized that increased risk may need further monitoring.

P7: I wouldn't want to be labelled as gestational diabetes, but I would want to know and I'd want a couple of extra checks, just to make sure everything's going okay

P6: I think you'd get that if you had raised blood sugar in pregnancy

P2: They would keep an eye on it, yeah

P6: Yeah

P4: You would get the same outcomes, but without the label and the stress

## 4 DISCUSSION

While the participants in our pilot community jury initially agreed that the most important consequence of a GDM diagnosis (rated as highest priority) was the ‘opportunity to minimize the risks to the unborn baby’, after reviewing the level of risks and upon reflection, the women changed their opinions and countered the ‘*knee jerk’* (day 2 pg 133) reaction they believe they had on day 1. After private deliberation, the women rated the most important consequence of a diagnosis of GDM to be the negative emotional state of the mother. The priority placed on this consequence over the physical consequences preferenced by the HAPO study,^1^ and subsequent definition GDM potentially suggests a significant pivot away from traditional clinical thinking.

Also challenging the traditional nomenclature of clinicians, the CJ participants grappled with what labels to ascribe the four different clinical states of GDM (all currently labelled as ‘GDM’). They recognized that the different clinical states warranted different labels to signal appropriate risks while mitigating unnecessary stress and burden on diagnosed women, which is reflected in the various labels they suggested for scenarios depicting different levels of GDM risk.

The women of this CJ clearly understood the numerical relative risks of physical consequences and the qualitative risks and benefits of other women's lived experiences. When informed of the potential risks and benefits of a diagnosis of GDM, women prioritize these consequences differently from clinicians. They also understood the different clinical states of GDM and concluded they differed sufficiently to warrant different ‘labels’. This pilot CJ had a small sample of women and should be repeated to explore whether women in other areas have similar or divergent views. However, the voices of informed, affected publics are often missing from panels that define health conditions and may differ from those of the clinicians. Their voices, preferences and opinions should be embedded in the decision‐making process.

We strengthened our methods by standardizing expert presentations as voice‐over PowerPoint to minimize presenter bias and providing hyperlinks to these in the article for transparency. A steering committee was established to develop the CJ questions, and members of this group conducted the presentations. Although a small sample, we included the voices of rural and regional women. As the women were all from South East Queensland, Australia, women from other regions might have different responses. We also represented the evidence of consequences of GDM by developing infographics of published and aggregated data^1^, ^22^ but recognize that women relied on this evidence to form their recommendations, and these may be different if other evidence were presented.

The adverse risk of perinatal outcomes used in the HAPO study^1^ underpinned the change in blood glucose levels used to diagnose GDM in some countries, such as Australia where the definition of GDM was changed in 2014. However, this change has been contentious, as since the change, some studies have reported increasing prevalence of GDM but few improvements in health outcomes.^10^, ^11^ Moreover, many women report emotional and financial challenges when diagnosed with GDM.^22^ As a risk factor, higher levels of hyperglycaemia are associated with increases the risk of post‐partum type 2 diabetes and cardiovascular disease, as well as increasing in the risk of adverse perinatal outcomes. However, the current definition of GDM in Australia is not stratified for risk and the women diagnosed with GDM (irrespective of risk) are treated similarly.

Our findings lend support to a reconsideration of GDM threshold definition in Australia and what different clinical states might be called. However, these finding should be interpreted in the context of deliberative methods and their strengths and limitations. CJs are small by design,^12^, ^13^, ^14^, ^15^ and this has sometimes been criticized. A scoping review of public deliberative techniques in health revealed CJs were the most common technique and ranged in size from 9 to 16 people.^23^ However, we had fewer participants than we anticipated, and the findings should be interpreted cautiously. We cannot claim that women's views from this CJ represent broader community views. Because we wanted to recruit the population most affected by the question of GDM definition, we recruited mothers, who are challenging to recruit as they experience many competing demands on their time. When contacted, many women could not attend the required 2 days, and some had sick children during the CJ weekend. Recruitment limitations from this study serve as key learnings, and future CJs on GDM will be adapted to suit the demands of young families. For example, for young mothers, it may be more prudent to conduct CJs on weekdays when childcare may be more accessible or conduct the CJ over multiple days/weeks (1 day a week for 2 weeks). Despite recruitment challenges, the women who did attend produced consensus recommendations and transcripts reflecting cohesive group discussion and no dissention. However, we upheld robust CJ methods. We recruited from the population we considered most affected by the question, and the jury verdict was unanimous.

## AUTHOR CONTRIBUTIONS

PG and RT conceived the project design. All authors provided project assistance in preparation and conduct. AMS and RT extracted, analysed and interpreted the data. All authors contributed to the drafting of the manuscript and approve the final version.

## Data Availability

The data that support the findings of this study are available from the corresponding author upon reasonable request.
