# Supplementary material for: Exploring women’s priorities for the potential consequences of a gestational diabetes diagnosis: A pilot community jury
Source: Health Expect. 2020 Feb 23;23(3):593–602. doi: 10.1111/hex.13036 (PMC7321745; doi:10.1111/hex.13036)
Supplement: Supplementary file 1 [file HEX-23-593-s001.pdf]

Supplementary File 1. A series of 'flash cards' outlining the published consequences of a GDM diagnosis provided to the CJ participants

Negative emotions such as self-blame, sadness and anxiety

Nearly all women expressed at least one negative emotion such as self-blame, sadness, feelings of failure, being scared, having concerns and being confused.

'I was very surprised and very upset to be diagnosed. I felt a little bit of a failure.'

'You actually feel guilty, right? Because this baby hasn't asked for this; and what if the baby comes out and has some kind of disease? Then it's my fault.

Source: Systematic review of qualitative research evidence, 2019

Opportunity to improve lifestyle

Some women received the diagnosis positively and said it was an opportunity to improve their diet and to learn about healthy eating.

'Wake-up call'

'Ever since I was diagnosed I've been eating healthy and I've been exercising, but it actually made me feel good.'

Source: Systematic review of qualitative research evidence, 2019[1]

‘Big baby’ i.e. birth weight $>90^{th}$ percentile

Having a large for gestational age (i.e. big baby) is an adverse pregnancy outcome for women with GDM.

Out of 100 women

not diagnosed with GDM

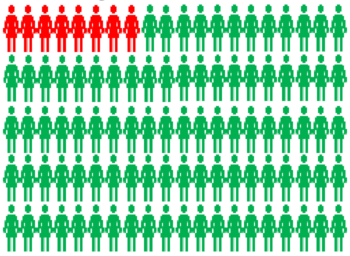

8 women will have a big baby and 92 women will not.

diagnosed with GDM

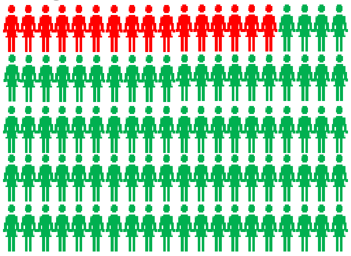

16 women will have a big baby and 84 women will not.

<u>This is a difference of 8 babies.</u>

Source: Hyperglycemia and Adverse Pregnancy Outcome study cohort [2,3]

Preterm Birth

Preterm birth is an adverse pregnancy outcome for women with GDM.

Out of 100 women

not diagnosed with GDM

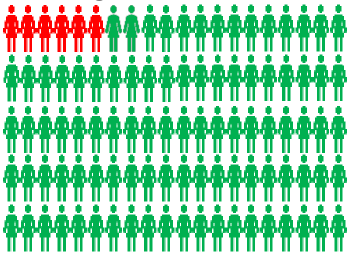

6 women will have a preterm birth and 94 women will not.

diagnosed with GDM

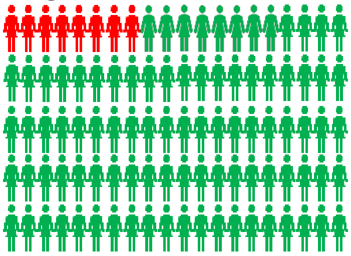

9 women will have a preterm birth and 91 women will not.

<u>This is a difference of 3 preterm births.</u>

Source: Hyperglycemia and Adverse Pregnancy Outcome study cohort [2,3]

The burden of managing GDM

Most women felt that with a diagnosis of GDM came with extra responsibility such as strict adherence to a dietary regime, activity restrictions, intense scheduling and frequent medical appointments which had an impact of their daily life.

Some women reported it took away the ‘joy of pregnancy’.

‘The whole pressure with the whole everything, it really did affect me and I think it’s probably one of the worst times I’ve had in my life actually’

‘It’s unreal. I never imagined that it would be this much work.’

Source: Systematic review of qualitative research evidence, 2019 [1]

Increased medical appointments

Nearly all women commented on having too many medical appointments to monitor their pregnancy.

‘It makes your life difficult if you have a demanding job very difficult because I would have some weeks where I’m having an ultrasound on Monday, I’m going to see the endocrinologist on Wednesday and the obstetrician on Thursday. So I’m missing 3 days in a week and I know that’s what our healthcare system is, that you go in and you sit there for 3 hours’

Source: Systematic review of qualitative research evidence, 2019 [1]

Over-medicalised pregnancy

Often women were automatically booked in for a caesarean section without consultation or lived in fear of this occurring.

A **few women** believed that they were being too closely monitored and felt over-scrutinised.

'they were so focused on the immediate pregnancy problems; within a medical model...OK, if we don't get it under control, we will just put you on insulin... just a drug solution'

'I said to myself 'oh please God let it be negative, I don't want to go and see these people', because you can't miss them or they will chase you. They will chase you… [This] scare [sic] a lot of people...we all want to be free, do our own thing, it's not like some people telling us what to do'

Source: Systematic review of qualitative research evidence, 2019 [1]

Dietary management-related stress

Many women reported dietary management-related stress amongst the women interviewed.

'It is frustrating still when you watch your carbs, you portion it and your reading is still high, almost every day.'

'I've been doing everything right. My sugar is so unstable. I have eaten the right foods, exercised, and tested my glucose levels four times a day. When my insulin dosages are increased, I am more depressed. I feel worthless.'

'You'd think, okay, well this will be good; this will be fine for me to eat. Then I will check my sugars 2 hours later and it would not. I would be why? That's not okay. It was disappointing, and it was definitely stressful, like it was just really not fun.

Source: Systematic review of qualitative research evidence, 2019 [1]

Opportunity to minimise risks to unborn baby

All women were concerned about the potential adverse effects that GDM can have on their baby. Women were motivated to adhere to treatment for the sake of their baby.

"your only thought is for the baby and for a favourable pregnancy outcome"

'…my baby might die if I'm not on [a] diet'

"I do not think about myself. My baby is the most important. . . . I fear I affect my baby"

Source: Systematic review of qualitative research evidence, 2019 [1]

Opportunity to reduce women's risk of developing diabetes after pregnancy

A **few women** were grateful to be advised that they were at risk of developing diabetes in the future, and now had the chance to minimise the risk.

'You have an active role and you can take charge of what's going on rather than just roll along.'

'GDM was a hidden blessing for me... GDM can go away after you have the baby but diabetes is not so easily fixable …I am much more aware of need to prevent it'

Source: Systematic review of qualitative research evidence, 2019 [1]

Shoulder dystocia or birth injury

Shoulder dystocia is where one of the baby's shoulders becomes stuck behind the mother's pelvic bone during childbirth.

Out of 100 women

not diagnosed with GDM

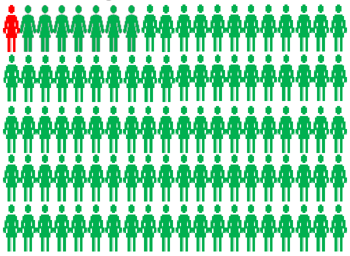

1 woman will have a baby with shoulder dystocia/ birth injury and 99 women will not.

.

diagnosed with GDM

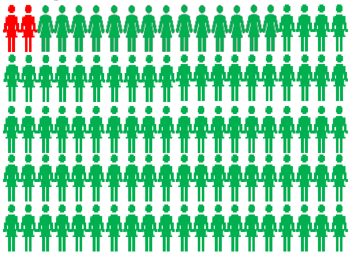

2 women will have a baby with shoulder dystocia/birth injury and 98 women will not.

<u>This is a difference of 1 baby with shoulder dystocia/birth injury.</u>

Source: Hyperglycemia and Adverse Pregnancy Outcome study cohort [2,3]

Caesarean section

A caesarean section is a surgical procedure to deliver a baby through a cut in the mother's abdomen (tummy) and uterus (womb).

Out of 100 women

not diagnosed with GDM

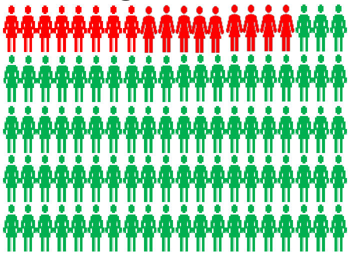

17 women will have a caesarean section and 83 women will not.

diagnosed with GDM

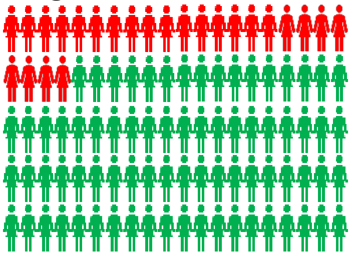

24 women will have a caesarean section and 76 women will not.

<u>This is a difference of 7 women.</u>

Source: Hyperglycemia and Adverse Pregnancy Outcome study cohort [2,3]

Other?

Other?

References

1. Blinded for review. Women's experiences of a diagnosis of gestational diabetes mellitus: a systematic review. Submitted to BMC Pregnancy and Childbirth, June 2019
2. McIntyre HD, Dyer AR, Metzger BE. Odds, risks and appropriate diagnosis of gestational diabetes. *Med J Aust* 2015;202(6):309-+. doi: 10.5694/mja14.01341
3. Metzger BE, Lowe LP, Dyer AR, et al. Hyperglycemia and adverse pregnancy outcomes. *N Engl J Med* 2008;358(19):1991-2002.
