# Supplementary material for: Exploring women’s priorities for the potential consequences of a gestational diabetes diagnosis: A pilot community jury
Source: Health Expect. 2020 Feb 23;23(3):593–602. doi: 10.1111/hex.13036 (PMC7321745; doi:10.1111/hex.13036)
Supplement: Supplementary file 2 [file HEX-23-593-s002.pdf]

Supplementary file 2. Descriptions of clinical states of GDM presented to the CJ participants

A: Developed diabetes as a result of pregnancy

Katie is 30 years old woman and 28 weeks pregnant and has undergone a blood sugar test. The results showed that she had a 2-hour blood sugar level of 12.1 (high).

Katie meets the criteria for (non-pregnant) diabetes. She did not meet the criteria before she was pregnant but does now.

B: Has higher than usual blood sugar levels as a result of the pregnancy and is at increased risk of complications

Jenny is 30 years old woman and 28 weeks pregnant and has undergone a blood sugar test. The results showed that she had a 2-hour blood sugar level of 9.0 (elevated).

Jenny does not fulfill the criteria for (non pregnant) diabetes but has blood sugar levels above normal due to the pregnancy. *Her higher than normal blood sugar levels increase the risk of complications*.

C: Has higher than usual blood sugar levels as a result of the pregnancy and is at normal risk of complications

Emily is 30 years old and 28 weeks pregnant and has undergone a blood sugar test. The results showed that she had a 2-hour blood sugar level of 8.4.

She has somewhat higher than usual blood sugar levels but *she is at normal risk of complication.*

D: Had diabetes before pregnancy and still has diabetes in pregnancy

Sofia is 30 years old and 28 weeks pregnant and has undergone a blood sugar test. The results showed that she had a 2-hour blood sugar level of 10.5.

She was known to have diabetes before being pregnant, and her pregnancy has further raised her blood sugar.

Her diabetes increases the risk of complications.

List of potential labels in random order:

Diabetes

Diabetes due to pregnancy

Diabetes in pregnancy

Gestational diabetes

Raised blood sugar in pregnancy

Hyperglycaemia in pregnancy

Altered glucose metabolism in pregnancy

Pregnancy induced diabetes

Diabetes of pregnancy

Reduced tolerance to raised blood sugar in pregnancy

Pregnant

Diabetes limited to pregnancy

Diabetes	Diabetes due to pregnancy
Diabetes in pregnancy	Gestational diabetes
Raised blood sugar in pregnancy	Hyperglycaemia in pregnancy
Altered glucose metabolism in pregnancy	Pregnancy induced diabetes

Diabetes of pregnancy	Reduced tolerance to raised blood sugar in pregnancy
Pregnant	Diabetes limited to pregnancy
